# Efficacy of Anti-Interleukin-5 Therapy with Mepolizumab in Patients with Asthma: A Meta-Analysis of Randomized Placebo-Controlled Trials

**DOI:** 10.1371/journal.pone.0059872

**Published:** 2013-03-27

**Authors:** Yao Liu, Song Zhang, Dao-wei Li, Shu-juan Jiang

**Affiliations:** Department of Respiratory Medicine, Provincial Hospital Affiliated to Shandong University, Jinan, Shandong, China; Tehran University of Medical Sciences, Iran (Republic of Islamic)

## Abstract

**Background:**

Interleukin (IL)-5 is believed to be a key cytokine in eosinophil inflammatory infiltration in asthma. Previous clinical trials have evaluated the efficacy and safety of mepolizumab, a monoclonal antibody against IL-5, in patients with asthma. However, most of these studies were small, the conclusions were inconsistent, and the precise effects are therefore debatable.

**Methods:**

A meta-analysis of randomized placebo-controlled trials was conducted to evaluate the effect of intravenous infusion of mepolizumab on clinical outcomes in patients with asthma. Trials were searched in PubMed, Embase, Web of Science, Cochrane CENTRAL, Scopus, reviews, and reference lists of relevant articles. The outcome variables analyzed included eosinophil counts in blood and sputum, airways outcome measures, exacerbations, asthma control, and quality of life scores.

**Results:**

Seven studies met final inclusion criteria (total n = 1131). From the pooled analyses, mepolizumab significantly reduced eosinophils in blood (MD −0.29×10^9^/L, 95% CI −0.44 to −0.14×10^9^/L, *P* = 0.0001) and sputum (MD −6.05%, 95% CI −9.34 to −2.77%, *P* = 0.0003). Mepolizumab was also associated with significantly decreased exacerbation risk than placebo (OR 0.30, 95%CI 0.13 to 0.67, *P* = 0.004), and with a significant improvement in the scores on the Asthma Quality of Life Questionnaire (AQLQ) (MD 0.26, 95% CI 0.03 to 0.49, *P* = 0.03) in patients with eosinophilic asthma. There were no statistical differences between the groups with respect to FEV_1_, PEF, or histamine PC_20_ (all *P*>0.05)_,_ and a non-significant trend for improvement in scores on the Juniper Asthma Control Questionnaire (JACQ) (MD −0.21, 95% CI −0.43 to 0.01, *P* = 0.06) in the mepolizumab group was observed.

**Conclusions:**

Mepolizumab reduces the risk of exacerbations and improves quality of life in patients with eosinophilic asthma, but no significant improvement in lung function outcomes was observed. Further research is required to establish the possible role of anti–IL-5 as a therapy for asthma.

## Introduction

Eosinophilic inflammatory infiltration in the bronchial mucosa is considered a central event in the pathogenesis of asthma. Activated eosinophils secrete granular basic proteins that damage the bronchial epithelium and smooth muscle contraction, increase mucous secretion, and cause vasodilation [1]. Airway eosinophil has been linked to airway hyperresponsiveness [2], [3], asthma symptoms, and airway narrowing in animal models and humans [4].

Interleukin (IL)-5 is a key cytokine in eosinophil differentiation, maturation, recruitment and activation at sites of allergic inflammation [5], [6]. Clinical studies have shown an increase in IL-5 in bronchoalveolar lavage fluid (BALF) and bronchial biopsies in asthma [7], and the level of IL-5 in BALF and the bronchial mucosa correlated with disease severity [8], [9]. Thus, IL-5 inhibition may have a beneficial therapeutic effect in asthma by preventing eosinophil maturation, function, or migration into pulmonary tissue.

Several placebo-controlled clinical trials have evaluated the efficacy and safety of mepolizumab, a humanized monoclonal antibody against IL-5, in patients with asthma [10]–[16]. However, the sample sizes were relatively modest, and the results were not consistent. We carried out a systematic review of the literature to provide an overview of the relevant studies, and to evaluate the efficacy of administering mepolizumab on blood and sputum eosinophils, lung function, clinical exacerbations, asthma control, and asthma related quality of life in patients with varied types of asthma.

## Methods

We conducted a meta-analysis using the guidelines of the Cochrane Collaboration [17], and our findings are reported according to the Quality of Reporting of Meta-analysis statement [18].

### Search Strategy and Selection Criteria

Two reviewers (YL and SJJ) systematically searched PubMed, Embase, ISI Web of Science, Cochrane CENTRAL, and Scopus for articles published until January 2013. The following keywords were used in searching: “anti–interleukin-5” or “mepolizumab” or “monoclonal antibody”, combined with “asthma”. Reviews and reference lists of relevant articles were also screened for additional articles of interest. Language restrictions were not applied. Completed, published, randomized controlled trials (RCTs) investigating the effect of mepolizumab on eosinophil counts in blood or sputum and clinical outcomes in patients with asthma were selected.

From the title, abstract or descriptors, the literature search was reviewed independently to identify potentially relevant trials for full review. In addition, a manual search of references from reports of clinical trials or review articles was performed to identify relevant trials. From the full text using specific criteria, the two reviewers independently selected trials for inclusion. There was no disagreement, although it was planned that disagreements would be resolved by a third party adjudication. Attempts were also made to contact investigators for unpublished data.

### Outcome Measures

Objective analyses focusing on the following outcome variables were undertaken. These included changes from baseline of blood eosinophil counts (10^9^/L), sputum eosinophils counts (%), the forced expiratory volume in 1 second (FEV_1_) (L) or FEV_1%_ of predicted value (%), peak expiratory flow (PEF) (L/min), provocative concentration of histamine required to cause a 20% fall in FEV_1_ (histamine PC_20_) (mg/ml), asthma exacerbation rates (%), scores on the Juniper Asthma Control Questionnaire (JACQ) and the Asthma Quality of Life Questionnaire (AQLQ). The JACQ assesses daytime and nighttime symptoms and activity limitation on the basis of five questions that are scored on a scale of 0 to 6, with lower numbers representing better control of symptoms [19]. The AQLQ is a 32-item questionnaire for patients with asthma that contains items in four domains (symptoms, emotions, exposure to environmental stimuli, and activity limitations), which is scored on a scale of 1 to 7, with higher scores indicating better asthma-related quality of life [20]. Studies that did not mention a specific outcome (or variable) were excluded from the analyses for this endpoint. If two or more assessment measures with different follow-up for an outcome were reported in one study, the outcome measure with the most common follow-up among the included studies was used for analysis.

### Data Items

Data extraction and critical appraisal were carried out by 3 reviewers (YL, SJJ, and SZ). Standardized data extraction forms [17] were used by these authors to independently and blindly summarize the RCTs meeting the inclusion criteria. The authors were not blinded to the source of the document or to authorship for the purpose of data extraction. The data were compared and discrepancies were resolved by consensus. Data on first author’s last name, the publication year, study design, the sample size, study population, baseline characteristics, treatment regimen (dose of mepolizumab, duration of treatment), length of follow-up, and outcomes were extracted. The reported adverse events during the treatment phase were collected to assess the safety of mepolizumab infusion.

### Risk of Bias and Quality Assessment

Risk of bias for each study was assessed using the tool available in the RevMan software. Six components were assessed: (1) adequate sequence generation; (2) allocation concealment; (3) blinding; (4) incomplete outcome data addressed; (5) free of selective reporting; and (6) free of other bias. Studies included in the review underwent quality assessment and were entered into a ‘risk of bias’ table. The studies were classified into A: low risk of bias and each of the criteria was appropriate; B: medium risk of bias and most of the criteria were appropriate; and C: high risk of bias and most of the criteria were not appropriate.

Jadad’s scoring system was also introduced to evaluate the quality of the studies [21]. Trials scored one point for each area addressed in the study design (randomization, blinding, concealment of allocation, reporting of withdrawals, and generation of random numbers) with a possible score of between 0 and 5 (highest level of quality). Higher numbers represented a better quality (Jadad’s score≥4).

### Statistical Analyses

Our meta-analysis and statistical analyses were performed with Revman software (version 5.0; Cochrane Collaboration, Oxford, United Kingdom) and Stata software (version 11.0; Stata Corporation, College Station, TX, USA), using odds ratios (ORs) for binary outcomes and mean differences (MDs) for continuous outcome measures. To pool continuous data, net changes in each of the study variables, which were calculated from baseline and follow-up means and SDs (follow-up minus baseline) were used to estimate the principle effect. When SDs were not directly available, they were calculated from SEs or CIs. For trials in which variances for paired differences were separately reported for each group, a pooled variance for the net change was calculated and the change-from-baseline SDs were computed by using correlation coefficient methods referenced in the *Cochrane Handbook for Systematic Reviews of Interventions* [18].

Random effect models, developed using the inverse variance weighted method approach, were used to combine the data.

Statistical heterogeneity of treatment effects between studies was formally tested with Cochran’s χ^2^ statistics and with significance set at *P*<0.10. The I^2^ statistic was used to quantify heterogeneity. Using accepted guidelines [18], an I^2^ of 0% to 40% was considered to exclude heterogeneity, an I^2^ of 30% to 60% to represent moderate heterogeneity, an I^2^ of 50% to 90% to represent substantial heterogeneity, and an I^2^ of 75% to 100% to represent considerable heterogeneity. If substantial heterogeneity was identified, subgroup and sensitivity analyses were performed. Publication bias was assessed with funnel plots and the Begg’s test.

## Results

### Literature Search and Study Characteristics

**Figure 1:**
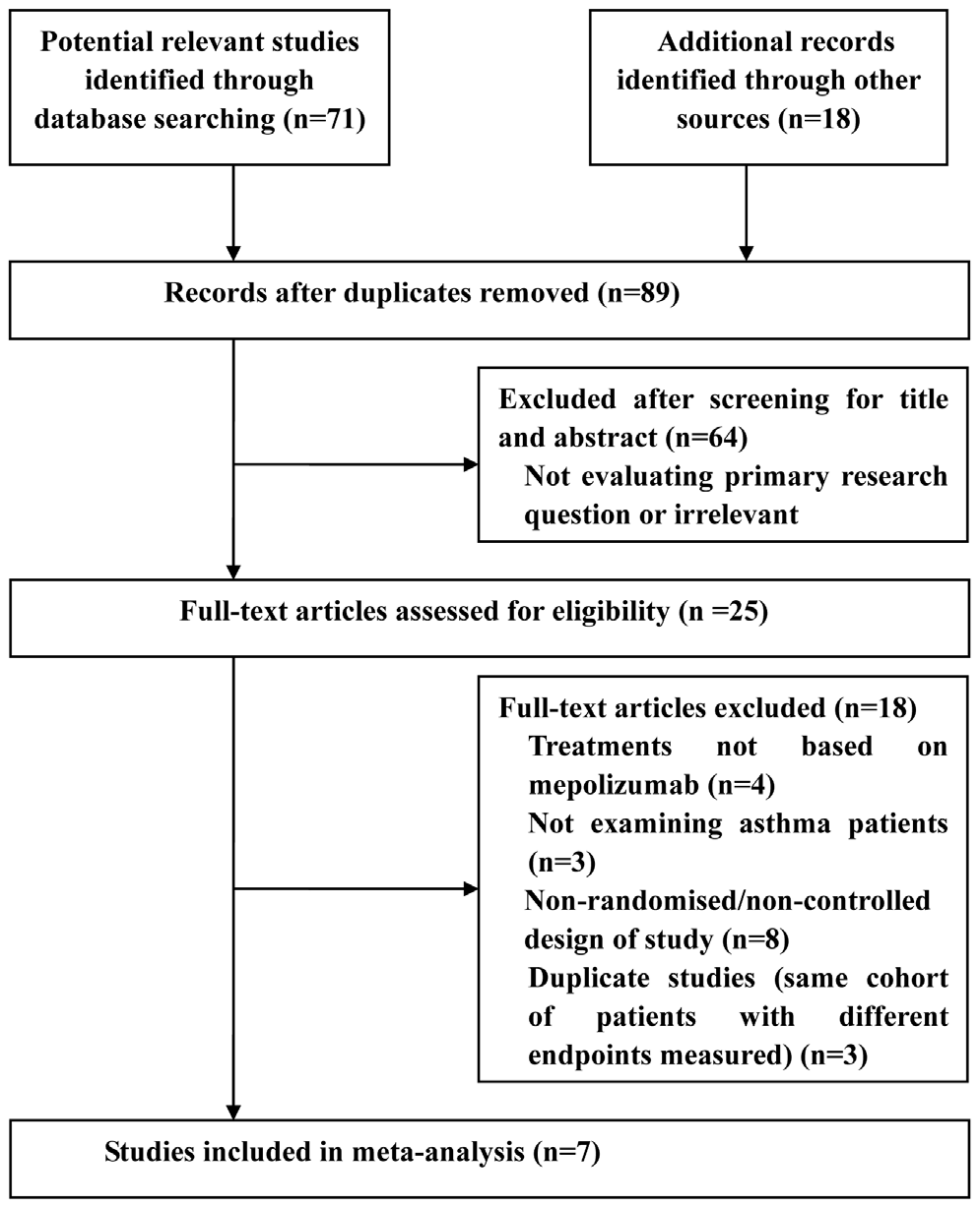
Flow of study identification, inclusion, and exclusion.

The method used to select studies is shown in Figure 1. A total of 89 potentially eligible articles were initially identified, and 64 articles were excluded as they were not relevant to the purpose of the current meta-analysis. Therefore, 25 potentially relevant articles were selected for detailed evaluation. From the overall pool of full-text articles, 18 articles were excluded because they were not based on mepolizumab treatment (n = 4), did not evaluate asthma patients (n = 3), were non-randomized/non-controlled studies (n = 8), or were duplicate studies (same cohort of patients with different endpoints measured) (n = 3).

We identified 7 RCTs [10]–[16] with 1131 subjects for inclusion in our study. Characteristics of the trials included are shown in Table 1. All of the 7 RCTs were double-blind, and placebo-controlled; 2 were single-centre studies and 5 were multi-centre studies. The trials varied in size from 19 to 621 subjects. The subjects were patients with mild atopic asthma in 2 studies [10], [12], mild or moderate asthma in 2 studies [11], [13], and eosinophilic asthma in the other 3 studies [14]–[16]. The mean age of the patients varied from 28 to 57 years. The duration of treatment ranged from 1 day to 52 weeks and follow-up ranged from 16 to 52 weeks. Participants received intravenous mepolizumab 750 mg in 3 studies, 250 mg or 750 mg in 2 studies, 2.5 mg/kg or 10 mg/kg in 1 study, and 75 mg, 250 mg, or 750 mg in 1 study. As 750 mg was the most common dose among the studies, we analyzed the effects of mepolizumab 750 mg (or 2.5 mg/kg in 1 study [10]) on all above outcomes in this meta-analysis. The study qualities of the selected trials were diverse, 4 trials [13]–[16] were classified as high quality (Jadad score ≥4) and 3 trials [10]–[12] were low quality (Jadad score of 2 or 3).

**Table 1:** Characteristics of 7 included randomized controlled trials *.

Author	Year	Study Design	No. ofSubjects(M/F)	Population	Meanage	Description of interventionand control arms	Outcomes	Treatment duration	Follow-up	Jadad’sscore
Leckie et al, 10	2000	Multi-center,double-blind,	24 (24/0)	Mild atopic asthma	28	One dose of mepolizumab 2.5 mg/kg or 10 mg/kg or placebo on day 1	Blood and sputum eosinophils; FEV_1_%; histamine PC_20_;	One day	16-wk	3
Bűttner,11	2003	Multi-centre double-blind,	19 (7/12)	Mild or moderate asthma	41	Mepolizumab 250 mg or 750 mg or placebo at intervals of 4 wk	Blood eosinophils;	3-mo	6-mo	2
Flood-Page PT et al, 12	2003	Two-center, double-blind,parallel	24 (17/7)	Mild atopic asthma	30	Three doses of mepolizumab 750 mg or placebo at intervals of 4 wks	Blood and bronchial eosinophils; FEV_1_%; PEF; histamine PC_20_;	8-wk	20-wk	3
Flood-Page P et al,13	2007	Multi-center,double-blind,	362 (140/202)	Moderate persistent asthma	38	Three doses of mepolizumab 250 or 750 mg, or placebo at intervals of 1 mo	Blood and sputum eosinophil; FEV_1_, PEF; symptom scores; and asthma exacerbation†.	12-wk	20-wk	4
Nair et al,14	2009	Single-cente,double-blind, pilot study	20 (12/8)	Eosinophilic asthma	57	Five doses of mepolizumab 750 mg or placebo at intervals of 1 mo	Blood and sputum eosinophils; asthma exacerbations‡; FEV_1._	16-wk	24-wk	5
Haldar et al, 15	2009	Single-center double-blind,parallel	61 (32/29)	Eosinophilic asthma with recurrent exacerbations	49	Twelve doses of mepolizumab 750 mg or placebo at intervals of 1 mo	Blood and sputum eosinophil; JACQ; AQLQ; FEV_1_; histamine PC_20_; asthma exacerbations§.	50-wk	50-wk	4
Pavord et al,16	2012	Multi-center,double-blind,	621 (NR)	Eosinophilic asthma with recurrent exacerbation	49	Thirteen doses of mepolizumab 75 mg, 250 mg, or 750 mg placebo at intervals of 4 wks	Blood and sputum eosinophil; asthma exacerbations¶; FEV_1_; JACQ scores; AQLQ.	52-wk	52-wk	5

* FEV_1_, forced expiratory volume in 1 second; PEF, peak expiratory flow; histamine PC_20_, provocative concentration of histamine required to cause a 20% fall in FEV_1_, asthma exacerbation rates; JACQ, Juniper Asthma Control Questionnaire; AQLQ, the Asthma Quality of Life Questionnaire; NR, not reported;

† An asthma exacerbation was defined as an acute worsening of asthma requiring additional treatment in excess of an increase in short-acting.

β_2_-agonist.

‡ Exacerbations were defined as increase in the daily dose of albuterol to control symptoms of chest tightness or as any one of the following: nocturnal or waking respiratory symptoms on two consecutive days, a decrease of more than 15% in the FEV_1_, or a 2-point worsening in the Likert score.

§ Exacerbations were defined as periods of deterioration in asthma control in subjects who had been treated with high-dose oral prednisolone for at least 5 days.

¶ Exacerbations defined as worsening of asthma requiring use of oral corticosteroids for 3 or more days, admission, or a visit to the emergency department–were corroborated by another measurement: decreased peak flow, 50% increase in rescue medication, increased frequency of nocturnal awakening due to asthma, or overall asthma symptom score of five for at least 2 of 3 successive days.

### Outcomes and Synthesis of Results

#### Blood and sputum eosinophil counts

**Figure 2:**
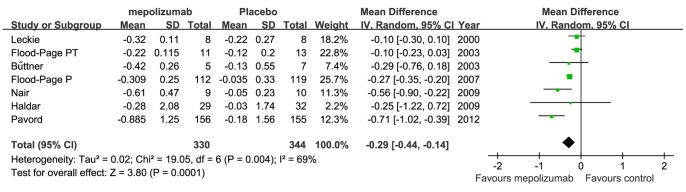
The effect of mepolizumab on blood eosinophils (×10^9^/L).

All the 7 studies determined the effect of mepolizumab on blood eosinophil counts [10]–[16] (Figure 2). Total sample sizes for mepolizumab and control group were 330 and 344, respectively. The pooled analysis showed infusion of mepolizumab was associated with a significant reduction in blood eosinophils (MD −0.29×10^9^/L, 95% CI −0.44 to −0.14×10^9^/L, *P* = 0.0001) compared with placebo. Statistical heterogeneity was observed among the studies (heterogeneity Chi^2^ = 19.05, I^2^ = 69%; *P* = 0.004).

**Figure 3:**
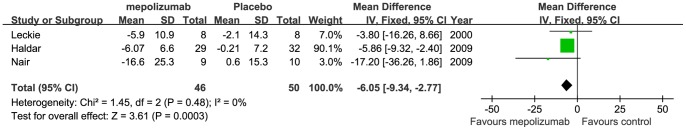
The effects of mepolizumab on sputum eosinophils (%).

The results for sputum eosinophils were reported in 3 studies [10], [14], [15] that represented 46 patients treated with mepolizumab and 50 with placebo. The use of mepolizumab was also associated with a significant decrease in sputum eosinophils (MD −6.05%, 95% CI −9.34 to −2.77%, *P* = 0.0003), and heterogeneity was not shown for this outcome (I^2^ = 0%, *P* = 0.48) (Figure 3).

#### FEV1 or FEV1% of predicted value

**Figure 4:**
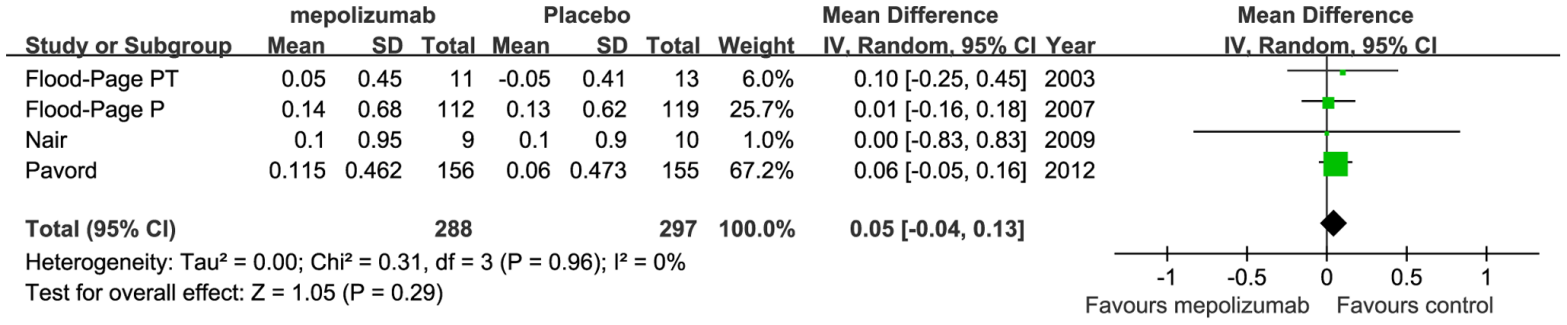
The effects of mepolizumab on FEV_1_ (L).

**Figure 5:**
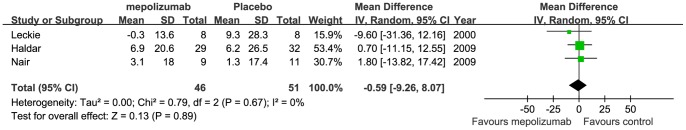
The effects of mepolizumab on FEV_1%_ of predicted value.

Four studies assessed the responsiveness of FEV_1_ or FEV_1_% of predicted value to treatment with mepolizumab [10], [12]–[16] (Figure 4 and 5), included 334 patients treated with mepolizumab and 348 with placebo. No significant differences were observed between mepolizumab and placebo group in changes from baseline values of FEV_1_ (MD 0.05 L, 95% CI −0.04 to 0.13 L, *P* = 0.29) or FEV_1_% of predicted value (MD −0.59%, 95% CI −9.26 to 8.07%, *P* = 0.89). Statistical heterogeneity was not observed (I^2^ = 0%, *P* = 0.96 and I^2^ = 0%, *P* = 0.67 ).

#### Peak expiratory flow (PEF)

**Figure 6:**
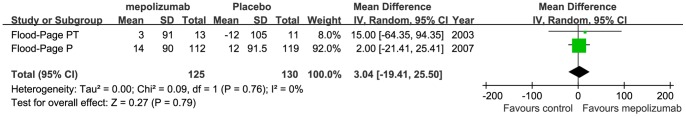
The effects of mepolizumab on morning PEF (L/min).

PEF was reported in 2 studies (255 patients) [12], [13] (Figure 6). Analyses of these studies showed a non-significant increase in PEF in the mepolizumab group compared with the placebo group (MD 3.04 L/min, 95% CI −19.41 to 25.50 L/min, *P* = 0.79). Heterogeneity was not found (I^2^ = 0%, *P* = 0.76).

#### Provocative concentration of histamine (histamine PC_20_)

**Figure 7:**
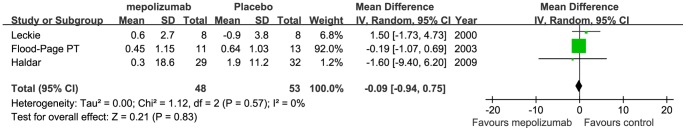
The effects of mepolizumab on histamine PC20 (mg/ml).

Estimates from 3 studies contributed to this analysis [10], [12], [15] (Figure 7). The pooled analyses showed there were no significant changes in histamine PC_20_ after treatment with mepolizumab compared with placebo (MD −0.09 mg/ml, 95% CI −0.94 to 0.75 mg/ml, *P* = 0.83). And statistical heterogeneity was not observed among these studies (I^2^ = 0%, *P* = 0.57).

#### Exacerbations

**Figure 8:**
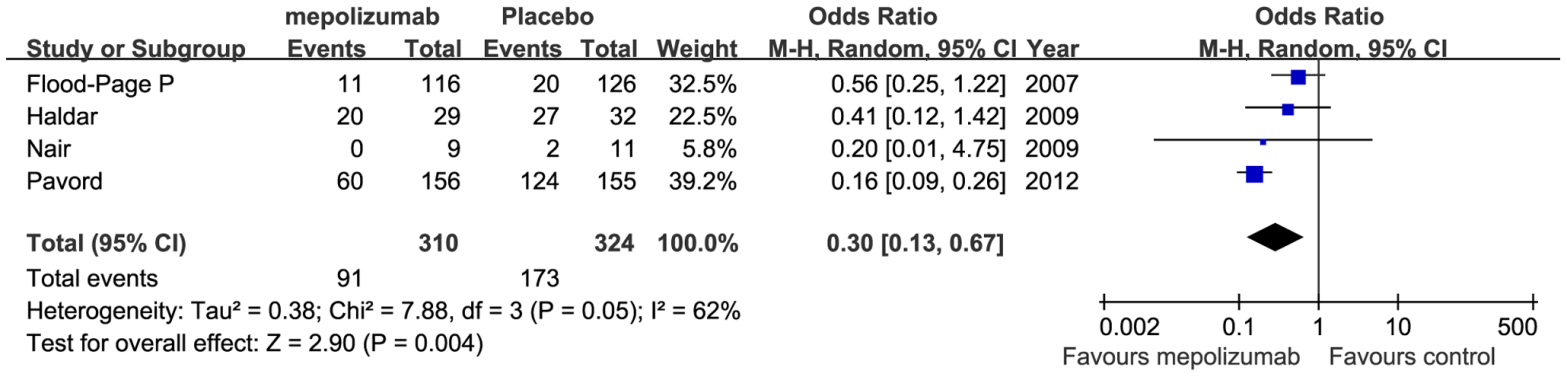
The effects of mepolizumab on exacerbation rates.

Four studies [13]–[16] evaluated if mepolizumab treatment reduced asthma exacerbation frequency. Sample sizes for mepolizumab and control groups were 310 and 324, respectively (Figure 8). Definitions for asthma exacerbation in original articles are summarized in Table 1. Although there were variations in these definitions, all the 4 studies defined exacerbation based on increase in the dose of corticosteroids or albuterol to control symptoms and/or deterioration in lung function. Analysis of these studies showed a higher proportion of patients in the placebo group (173 of 324; 53.4%) had exacerbations during the study period, compared with the mepolizumab group (91 of 310; 29.3%). From the pooled analysis, mepolizumab treatment was associated with significantly decreased risk of exacerbation (OR 0.30, 95%CI 0.13 to 0.67, *P* = 0.004). And statistical heterogeneity was shown between studies (I^2^ = 62%, *P* = 0.05).

#### Asthma control and Quality of Life Assessment

**Figure 9:**
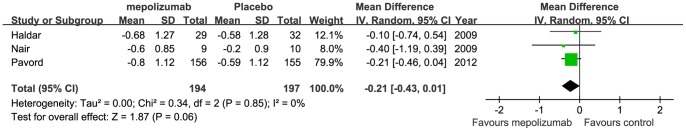
The effects of mepolizumab on Juniper Asthma Control Questionnaire (JACQ).

Three studies assessed asthma control with the use of JACQ [14]–[16] (Figure 9). The pooled analysis showed mepolizumab was associated with a non-significant improvement in scores on the JACQ (MD −0.21, 95% CI −0.43 to 0.01, *P* = 0.06). No statistically significant heterogeneity was observed between studies (I^2^ = 0%, *P* = 0.85).

**Figure 10:**
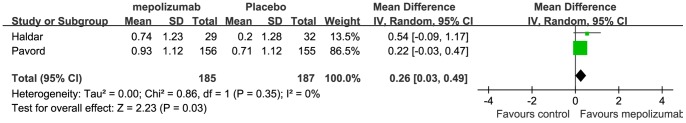
The effects of mepolizumab on Asthma Quality of Life Questionnaire (AQLQ).

Quality of life was assessed in 2 studies with the use of the AQLQ [15], [16] (Figure 10). Findings from the meta-analysis showed a greater improvement was observed in the AQLQ score in the mepolizumab group as compared with the placebo group (MD 0.26, 95% CI 0.03 to 0.49, *P* = 0.03). The χ2 test for heterogeneity was also non-significant (I^2^ = 0%, *P* = 0.35).

### Risk of Bias in Individual Studies

**Figure 11:**
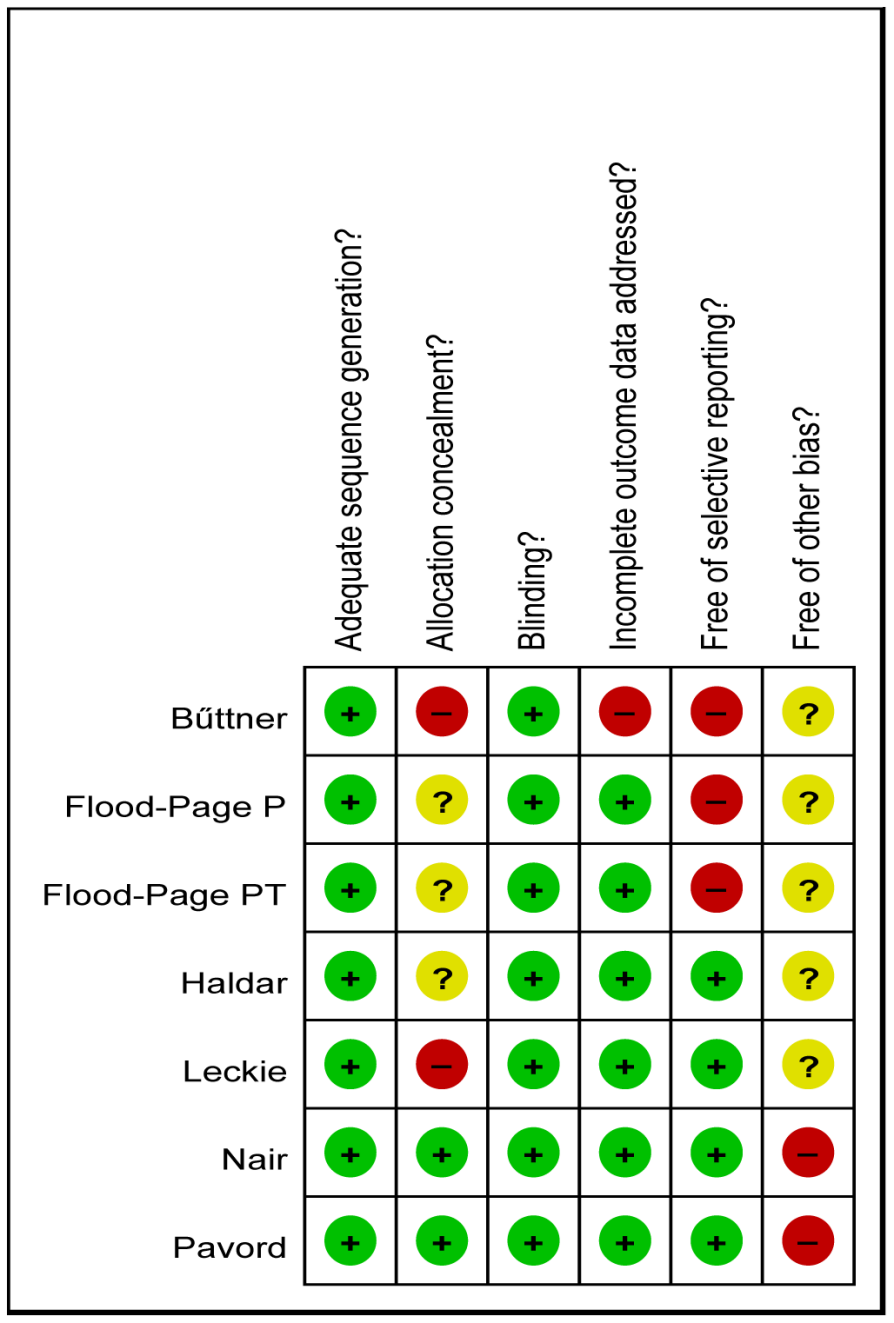
Risk of bias summary of included studies summary*. *Review authors’ judgments about each risk of bias item for each included study.+is “yes”, 2 is “no”,? is “unclear”.

Figure 11 provides a summary of methodological domain assessments for each including study. The study populations in all 7 trials were randomly allocated [10]–[16]. The randomization techniques were mentioned in 4 trials, including computer-generated randomization codes, 1∶1 ratio and minimization method [10], [11], [14], [16]. All the 7 studies were described as being double-blinded. Allocation concealment was adequate in only 2 studies [14], [16]. Incomplete outcome data were adequately addressed in 6 studies [10], [12]–[16]. And in 3 studies, some outcome measures were recorded but not all were reported [11]–[13].

### Safety

Mepolizumab was well tolerated. Some serious adverse events reported such as cerebrovascular disorder, asthma exacerbation and gastrointestinal disturbance were not considered by the investigators to be related to study medication. The common adverse events were as follows: headache, chest pain, facial flushing, erectile or ejaculatory dysfunction, rash, conjunctivitis, fatigue, upper respiratory tract infection, rhinitis, bronchitis, sinusitis, viral infection, injury, nausea, and pharyngitis.

### Subgroup and Sensitivity Analyses

To clarify the heterogeneity, subgroup analyses were carried out for blood eosinophils and asthma exacerbations. The results are shown in Table 2. The studies were stratified according to the number of subjects, types of asthma, mepolizumab administration frequency and the duration of follow-up. Analyses showed the efficacy of mepolizumab on blood eosinophils or asthma exacerbations were not influenced by the sample size, administration frequency or follow-up duration, except for types of asthma. A greater reduction effect in blood eosinophils was observed in patients with eosinophilic asthma compared to other asthma phenotypes (*P* for subgroup difference = 0.0008), and also a greater decrease in the risk of exacerbations was shown in those with eosinophilic asthma (*P* for subgroup difference = 0.02). Sensitivity analysis that excluded low-quality studies [10]–[12] revealed no appreciable change in the final results for blood eosinophils.

**Table 2:** Subgroup analyses for the effect of mepolizumab on blood eosinophil counts and asthma exacerbation.

Variables	Blood eosinophil counts			Asthma exacerbation			
	No.of studies	OR (95% CI)	*P* for Subgroup difference	No.of studies		OR (95% CI)	*P* for Subgroup difference
**Subgroup analysis**							
No. of subjects			0.25				0.75
<100	5	−0.20 (−0.37, −0.03)			2	0.37 (0.12,0.98)	
≥100	2	−0.46 (−0.88, −0.04)			2	0.28 (0.08,0.98)	
Types of asthma			0.0008				0.02
Eosinophilic asthma	3	−0.62 (−0.84, −0.39)			3	0.18 (0.11, 0.29)	
Mild or moderate asthma	4	−0.18 (−0.30, −0.06)			1	0.56 (0.25, 1.22)	
Mepolizumab dosage			0.08				0.13
≤5 intravenous doses of 750 mg	4	−0.22 (−0.36, −0.07)			2	0.52 (0.24, 1.12)	
>5 intravenous doses of 750 mg	3	−0.53 (−0.83, −0.22)			2	0.21 (0.09, 0.52)	
Follow-up			0.10				0.13
<50-wk	5	−0.32 (−0.45, −0.09)			2	0.52 (0.24, 1.12)	
≥50-wk	2	−0.66 (−0.96, −0.46)			2	0.21 (0.09, 0.52)	
**Sensitivity analysis**			*P* for association				
High-quality studies(Jadad’s score≥4)	4	−0.46 (−0.73, −0.09)	<0.001		All 4 studies with Jadad’s score≥4		

### Publication Bias

**Figure 12:**
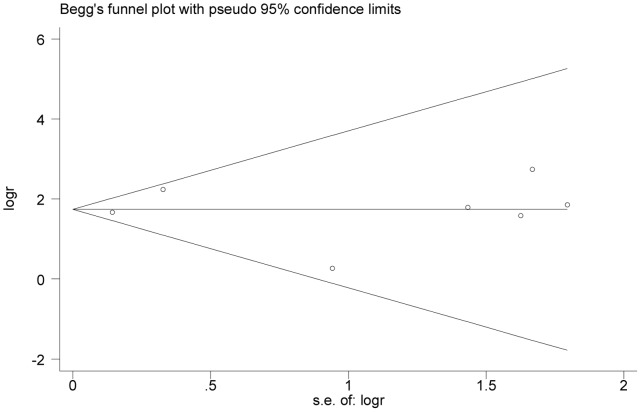
Begg’s funnel plot (with pseudo 95% CIs) of the 7 studies evaluated the effect of mepolizumab on blood eosinophils.

We performed funnel plot analysis and Begg’s test to assess publication bias. Funnel plot of the 7 studies evaluated the effect of mepolizumab on blood eosinophils appeared to be symmetrical through visual examination (Figure 12), and the Begg’s test of funnel plot suggested no publication bias (*P* = 0.95). And also no publication bias was detected by Begg’s test for other outcomes analysis (all *P*>0.05).

## Discussion

In the present study, we combined data that evaluated the efficacy of mepolizumab, a monoclonal antibody to IL-5, in patients with asthma. Based on 1131 asthma patients in 7 studies, we found mepolizumab significantly lowered blood and sputum eosinophil counts, effectively reduced asthma exacerbation frequency, and improved scores on the AQLQ versus placebo. In contrast, mepolizumab had no clinically significant effects on functional airway outcomes including FEV1, PEF, PC_20_, and a non-significant trend for a reduction in symptom scores assessed with JACQ was observed. Moreover, mepolizumab was well tolerated with minimal adverse events associated with drug administration.

Asthma is characterized by a prominent eosinophilic inflammatory infiltration in the bronchial mucosa [3]. Clinical studies have shown levels of eosinophils in peripheral-blood and BALF correlated with the clinical severity of asthma [4], suggesting that eosinophils may play a role in tissue remodeling events in patients with asthma. As IL-5 is a key cytokine in eosinophil differentiation and maturation in the bone marrow as well as in recruitment and activation at sites of allergic inflammation [22], IL-5 inhibition may have a beneficial therapeutic effect in asthma by preventing eosinophilic inflammation in pulmonary tissue. Our meta-analysis indicated that mepolizumab was significantly more effective in reducing blood and sputum eosinophils than placebo, which was in accordance with the results of previous studies involving patients with the hypereosinophilic syndrome [23].

However, our analysis did not demonstrate significant improvement in any of the functional airway outcomes (FEV1, PEF, and PC_20_). There are several possible explanations for the lack of observed benefit in lung function from mepolizumab treatment. Firstly, noneosinophilic or neutrophilic airway inflammation might contribute to persistent asthma symptoms in patients treated with inhaled corticosteroids, and such patients would be unlikely to respond to anti–IL-5 treatment [24]. Furthermore, although mepolizumab has marked effects in reducing blood eosinophils, the inability to completely abolish airway eosinophils also contributes to the lack of improvement in lung function outcomes [12]. Moreover, anti–IL-5 treatment had no effect on bronchial mucosal staining of eosinophil major basic protein, suggesting that reduction in eosinophil numbers does not reflect tissue deposition of granule proteins [12]. Therefore, tissue eosinophils may be less responsive to IL-5, making the elimination of IL-5 redundant. However, with the relatively small sample sizes and short follow-up duration of the included studies, the ability to draw conclusions is limited. Existing findings suggest measures of airway outcomes do not indicate improvements elicited by reduced eosinophilic airway inflammation, which have important implications for the choice of the outcomes in further clinical trials defining the potential utility of anti–IL-5 for asthma.

In contrast to the non-significant results in lung function outcomes, our meta-analysis showed a significant reduction in exacerbation rates for mepolizumab treatment compared with placebo. As exacerbations may differ from day-to-day symptoms in that they respond poorly to usual inhaled therapy and are more closely linked to increased airway inflammation [25], the link to eosinophilic inflammation may be particularly important. Several previous studies revealed that markers of eosinophilic airway inflammation increased well before the onset of exacerbations [26], [27]. In particular, Green and coworkers adjusted inhaled steroid dose according to sputum eosinophils and showed that this resulted in a dramatic reduction in exacerbation frequency [28]. These findings have been confirmed in a similar study in which monitoring sputum eosinophil counts was found to benefit patients with moderate-to-severe asthma by reducing the frequency and severity of exacerbations [24]. Our study also showed a significant improvement in asthma-related quality of life with mepolizumab therapy, perhaps reflecting the value to patients of the prevention of asthma exacerbations.

The different effects of mepolizumab in asthma exacerbations and lung function outcomes suggest a number of issues that need to be considered before this treatment approach administered. First of all, selection of the patient population might respond to anti–IL-5 is especially important. In the DREAM trial, Pavord et al investigated which baseline variable was associated with treatment response and identified only baseline blood eosinophils and exacerbation frequency in the previous year were associated with the efficacy of mepolizumab treatment [16]. This suggests that patients who could benefit from mepolizumab would be a population with high numbers of airway eosinophils, and repeated exacerbations, who are already taking and failing conventional treatments. Another issue with defining the potential utility of mepolizumab for asthma is the choice of the clinical outcomes might be associated with eosinophilic inflammation. The separation between airway outcomes and exacerbation risk implies that separate aspects of the disorder require different management strategies. Traditional markers of asthma such as FEV_1_ and the acute bronchodilator response may not be related to the efficacy of anti–IL-5, while existing data suggested the pathogenesis of asthma exacerbation appear to be correlated with eosinophilic inflammation [14]–[16].

### Limitations of the Review

Despite the intriguing results of the present meta-analysis, some potential limitations should be addressed. Firstly, this systematic review is limited to 7 studies with 1131 subjects. The sample size was not large enough to reach a convincing conclusion and could not be considered clinically directive. Secondly, the drug administration frequency and treatment duration differed in the trials involved in our meta-analysis, which made it difficult to determine the optimal dose of mepolizumab that would be mostly appropriate for patients with asthma. Thirdly, although these studies shared many common issues, there were also substantial heterogeneities across studies, notably the type of patients included, study design, follow-up duration, and definitions of asthma exacerbation. Given this limitation, the results should be interpreted cautiously. Moreover, inherent assumptions made for any meta-analysis, because the analysis pooled published data, and individual data or original data were unavailable, which restricted us doing more detailed relevant analysis and obtaining more comprehensive results.

### Conclusion

In conclusion, the current meta-analysis indicates that mepolizumab treatment appears to be useful for control of exacerbations and improve asthma-related quality of life in individuals with persistent airway eosinophilia, but may not associate with significant improvement in functional airways outcomes. The results highlight the importance of selection the subgroup of patients with asthma might derive clinical benefit from mepolizumab treatment. Additional larger studies will be required to establish the possible role of anti–IL-5 as a therapy for asthma.
