# Correction: Efficacy of Anti-Interleukin-5 Therapy with Mepolizumab in Patients with Asthma: A Meta-Analysis of Randomized Placebo-Controlled Trials

**DOI:** 10.1371/annotation/8da4be4b-2de1-4c51-9c40-0f49dc212579

**Published:** 2013-06-25

**Authors:** Yao Liu, Song Zhang, Dao-wei Li, Shu-juan Jiang

Figure 4 'The effects of mepolizumab on FEV1 (L)' in our article contains an error; the axis labels 'favours mepolizumab' and 'favours control' in the figure have been reversed. The authors apologize for this error and provide a revised Figure 4. The results are unaffected by this error, no significant differences are observed between mepolizumab and placebo group in changes from baseline values of FEV1.

Please see Figure 4 at the following link: 

**Figure:**
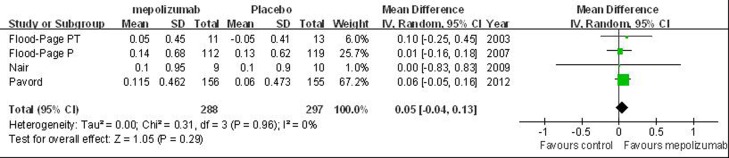


In addition, we wish to clarify that the meta-analysis only included analyses on the effects of a dose of 750 mg of mepolizumab on all outcomes. The reasons for this are as follows: in the seven randomized controlled trials included in this study, the mepolizumab dose was 750 mg in three studies, 250 mg or 750 mg in two studies, 2.5 mg/kg or 10 mg/kg in one study, and 75 mg, 250 mg, or 750 mg in one study. As 750 mg was the most common dose among the seven trials and the study data for other doses were too few to pool, we chose a dose of 750 mg of mepolizumab for the analyses. As a result, the data from the 75mg and 250mg doses were excluded from the analyses.

